# System identification: a feasible, reliable and valid way to quantify upper limb motor impairments

**DOI:** 10.1186/s12984-023-01192-x

**Published:** 2023-05-25

**Authors:** Mark van de Ruit, Levinia L. van der Velden, Bram Onneweer, Joyce L. Benner, Claudia J. W. Haarman, Gerard M. Ribbers, Ruud W. Selles

**Affiliations:** 1 Department of Biomechanical Engineering, Delft University of Technology, Mekelweg 2, 2628CD Delft, The Netherlands; 2 Department of Rehabilitation Medicine, Erasmus MC University Medical Center Rotterdam, Doctor Molewaterplein 40, 3015 GD Rotterdam, The Netherlands; 3 Rijndam Rehabilitation, Westersingel 300, 3015 LJ Rotterdam, The Netherlands; 4 Department of Biomechanical Engineering, University of Twente, Enschede, The Netherlands; 5 Hankamp Rehab, Enschede, The Netherlands

**Keywords:** Viscoelasticity, Biomechanics, Upper limb, Diagnostics, Motor impairments

## Abstract

**Background:**

Upper limb impairments in a hemiparetic arm are clinically quantified by well-established clinical scales, known to suffer poor validity, reliability, and sensitivity. Alternatively, robotics can assess motor impairments by characterizing joint dynamics through system identification. In this study, we establish the merits of quantifying abnormal synergy, spasticity, and changes in joint viscoelasticity using system identification, evaluating (1) feasibility and quality of parametric estimates, (2) test–retest reliability, (3) differences between healthy controls and patients with upper limb impairments, and (4) construct validity.

**Methods:**

Forty-five healthy controls, twenty-nine stroke patients, and twenty cerebral palsy patients participated. Participants were seated with the affected arm immobilized in the Shoulder-Elbow-Perturbator (SEP). The SEP is a one-degree-of-freedom perturbator that enables applying torque perturbations to the elbow while providing varying amounts of weight support to the human arm. Participants performed either a ‘do not intervene’ or a resist task. Elbow joint admittance was quantified and used to extract elbow viscosity and stiffness. Fifty-four of the participants performed two sessions to establish the test–retest reliability of the parameters. Construct validity was assessed by correlating system identification parameters to parameters extracted using a SEP protocol that objectifies current clinical scales (Re-Arm protocol).

**Results:**

Feasibility was confirmed by all participants successfully completing the study protocol within ~ 25 min without reporting pain or burden. The parametric estimates were good with a variance-accounted-for of ~ 80%. A fair to excellent test–retest reliability was found ($ICC = 0.46-0.98$) for patients, except for elbow stiffness with full weight support ($ICC = 0.35$). Compared to healthy controls, patients had a higher elbow viscosity and stiffness during the ‘do not intervene’ task and lower viscosity and stiffness during the resist task. Construct validity was confirmed by a significant (all $p<0.03$) but weak to moderate ($r = 0.36-0.50$) correlation with parameters from the Re-Arm protocol.

**Conclusions:**

This work demonstrates that system identification is feasible and reliable for quantifying upper limb motor impairments. Validity was confirmed by differences between patients and controls and correlations with other measurements, but further work is required to optimize the experimental protocol and establish clinical value.

**Supplementary Information:**

The online version contains supplementary material available at 10.1186/s12984-023-01192-x.

## Introduction

Upper limb motor impairments in hemiparetic limbs such as after stroke and cerebral palsy (CP) are currently assessed by a set of well-established clinical scales aiming to quantify, among others, muscle weakness, abnormal synergy, spasticity and changes in joint viscoelasticity [1]. These include tests like the Brunnstrom Fugl-Meyer, Modified Ashworth Scale (MAS), and Modified Tardieu Scale (MTS). The motor impairments identified using these tests assist in selecting a treatment approach and are used to monitor improvement. However, the tests have several known limitations, which include their poor validity, reliability, and sensitivity [2–6]. Whereas the need for objective and reliable assessment of motor impairments is widely recognized [7, 8], no tests have yet successfully replaced traditional clinical scales.

Robotics can objectively quantify the motor impairments of muscle weakness, abnormal synergy, spasticity, and changes in joint viscoelasticity [9–11]. Recently, a robotic device was developed and validated to assess these four impairments using one single device (the Shoulder-Elbow-Perturbator—SEP) [12]. By using the SEP to mimic current clinical tests, an earlier finding was confirmed that upper limb synergy can be quantified by the change in elbow range of motion (ROM) when gradually reducing arm weight support [13]. Likewise, spasticity and viscoelasticity can be objectively quantified by imposing passive joint movements at low and high speeds [9, 14, 15]. The robotics used in the aforementioned studies leveraged the accurate recording of joint angles and torques to provide a reliable way to quantify motor impairments during passive and active joint movements.

Motor impairments can also be quantified with the help of robotics by applying external mechanical perturbations while participants perform either a ‘do not intervene’ (DNI) or resist task [16, 17]. These tasks imply the participant is fully relaxed (DNI task) or attempts to maintain a constant joint angle or torque (resist task) while the joint is rotated within a small ROM (e.g., root mean square rotation of 0.5–1°). Recordings of the joint angle and joint torque are subsequently scrutinized using system identification techniques to characterize the joint dynamics. System identification enables us to quantify both the intrinsic (non-neural) and reflexive (neural) contribution to the joint dynamics [18, 19], captured in the joint’s inertia, viscosity, and stiffness parameters. These parameters can be translated to clinically described phenomena such as synergy (i.e., aberrant elbow stiffness when lifting the arm), spasticity (i.e., enhanced reflexive activity), or changes in viscoelasticity (i.e., increased intrinsic joint viscosity and stiffness).

In this study, we aim to establish the merits of quantifying abnormal synergy, spasticity, and changes in joint viscoelasticity using system identification. This is achieved by (1) confirming the feasibility to perform a system identification protocol and quality of parametric estimates, (2) establishing the test–retest reliability for the system identification parameters, (3) comparing obtained parameters between healthy controls and patients with upper limb impairments and (4) test construct validity by comparing the system identification parameters for each motor impairment with parameters obtained using four tests as part of a recently proposed robotic assessment protocol (Re-Arm) [12].

## Methods

### Participants

We recruited adult patients from the outpatient clinic of the Rijndam Rehabilitation Center that presented upper limb motor impairments due to stroke and CP. Patients were only included if they: (1) had a clinically-confirmed upper limb impairment, (2) were able to achieve active shoulder abduction (up to 80°), (3) were able to achieve visible active elbow extension, and (4) had a minimal passive range-of-movement for shoulder abduction of 80° and horizontal shoulder adduction of 45°. Exclusion criteria were: (1) hemiplegic shoulder pain, (2) a history of pre-existing neuromuscular disorders affecting upper limb function, (3) fixed upper limb contractures, and (4) the inability to understand verbal instructions. We only included stroke patients when they were considered chronic, suffering the stroke at least six months before study inclusion.

For comparison, we recruited a group of age-matched healthy controls without a known history of neurological or orthopedic disorders. The study was approved by the Medical Ethics Committee of the Erasmus Medical Center, Rotterdam, (protocol number NL64660.078.18), and conducted following the declaration of Helsinki.

### Experimental setup

#### Shoulder elbow perturbator

**Fig. 1:**
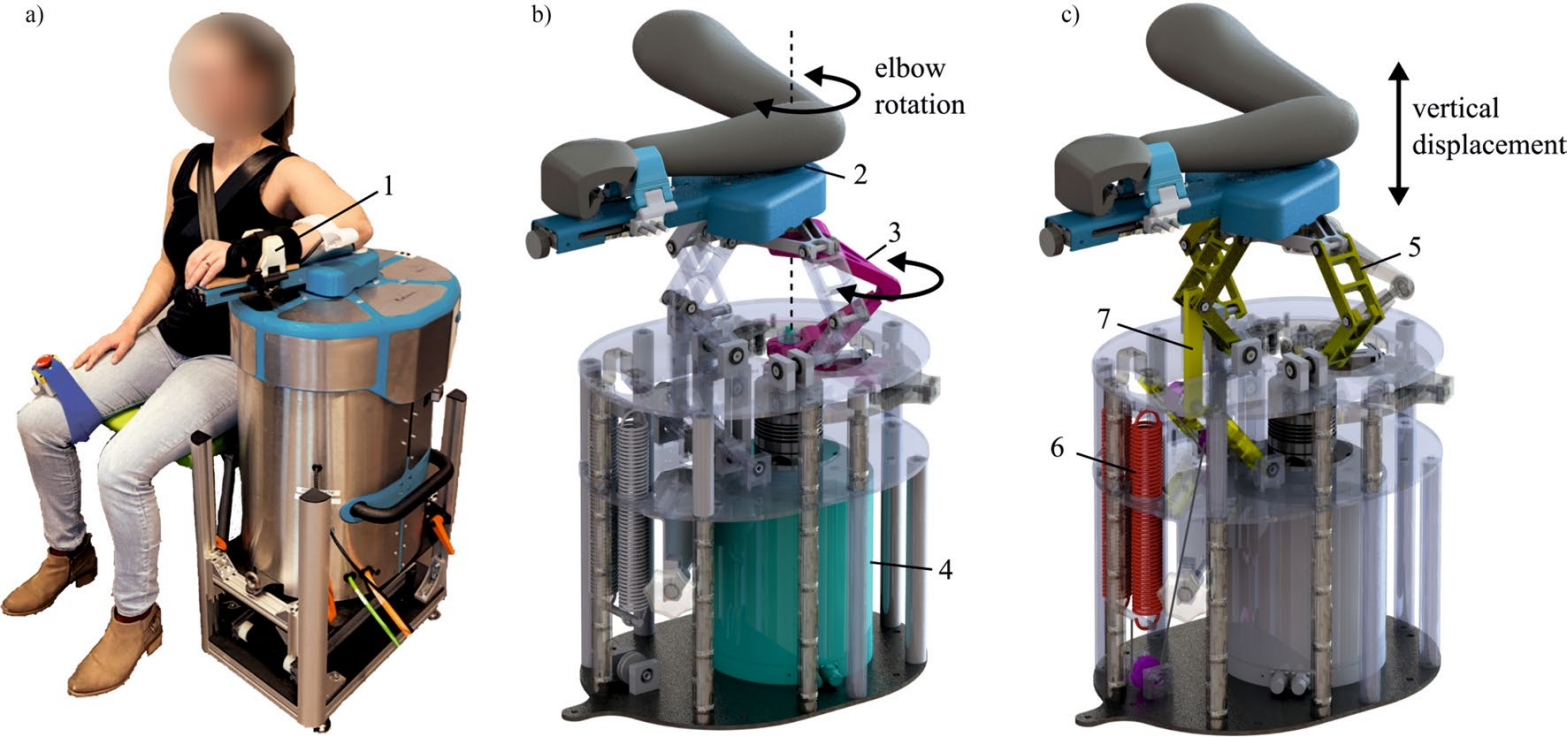
The Shoulder Elbow Perturbator (SEP), a robotic device to quantify multiple upper limb impairments. **a** Participant positioned in the SEP with the shoulder abducted in 80°, the forearm strapped (1) to the lever arm of the SEP, and the medial epicondyle of the humerus aligned with the motor rotation axis. **b** Internals of the SEP showing that the motor (4) transmits a torque through the torque link (3), allowing elbow rotation (2). **c** Internals of the SEP showing the shoulder abductor manipulation mechanism. The sarrus linkages (5) allow vertical displacement of the arm, and the arm is supported by two springs (6) with an upward force. With the cable routing and pulley configuration, the upward force can be manipulated independently of the linkage position (7). (Modified – with permission from [20])

The Shoulder-Elbow-Perturbator (SEP—Hankamp Rehab, Enschede, The Netherlands) was used to assess participants' elbow dynamics. The design of the SEP enables independent manipulation of elbow angle and weight support of the human arm [12]. A direct-drive servo motor (HIWIN TMS3C, Offenburg, Germany) attached to a lever supporting the lower arm controlled the elbow angle by aligning its axis to the medial epicondyle of the humerus (Fig. 1). A computer with Etherlab and MATLAB Simulink was used to control the SEP and capture elbow torque and angle with a sample rate of 1 kHz.

### Measurement protocol

All ninety-four participants underwent the same measurement protocol. Fifty-four participants performed the measurement protocol twice, separated by at least 7 days, to assess the test–retest reliability of the extracted parameters from each task.

#### Participant profiling

Before the SEP experiments, the age, sex, dominant arm, and side of the paretic arm were registered and body mass and length were measured. For stroke patients, the date and type of stroke were obtained from their medical records. For CP patients, the level of the Gross Motor Function Classification System (GMFCS) and the Manual Ability Classification (MACS) were obtained from their medical records [21, 22]. Finally, for each patient, synergy was quantified by either the score on the upper limb section of the Fugl-Meyer assessment (for stroke patients) or the Test of Arm Selective Control (TASC, for CP patients) [23, 24]. Spasticity was quantified using the MTS. All clinical tests were performed by an experienced assessor (LL).

#### SEP measurements

Participants were seated next to the SEP on a custom-made chair. Straps were used to limit torso displacement by immobilizing the torso against the back of the chair. The chair and the height of SEP were adjusted to achieve 80° shoulder abduction and 30° horizontal adduction. To ensure the axis of the servo motor remained aligned with the medial epicondyle of the humerus, the lower arm and wrist were fixed to the SEP lever using Velcro straps and a custom clamp mechanism with safety pins. The wrist remained in a ± 10° dorsiflexion position throughout the experiment using a cock-up cast. Before commencing the tests, the SEP ROM limits for the arm were determined manually.

Each participant was measured according to the ‘Re-Arm protocol’ [12] and the ‘System identification protocol’. All performed tests were presented in random order to avoid an order effect. Moreover, a 5-min rest period was adopted between each test to prevent fatigue.

##### System identification protocol

A system identification protocol was used as an all-encompassing way to quantify upper limb impairments using an active and passive elbow perturbation task. Participants were instructed to maintain the 80° shoulder abduction angle during the recordings during either a DNI or a resist task while receiving continuous torque perturbations to the elbow. The DNI task was performed once for five different arm weight support levels (100%, 75%, 50%, 25%, and 0% support). In contrast, the resist task was performed only once at two weight support levels (100 and 0% support) to limit fatigue and participant burden. The 100% weight support level was determined by gradually increasing the upward force of the support mechanism until the participant could fully relax the arm while the support surface remained at the same height. To aid participants in minimizing elbow movements during the resist task conditions, a vertical bar was mounted to the SEP near the hand to highlight the reference position where the hand had to be kept. Each trial lasted 55 s.

**Fig. 2:**
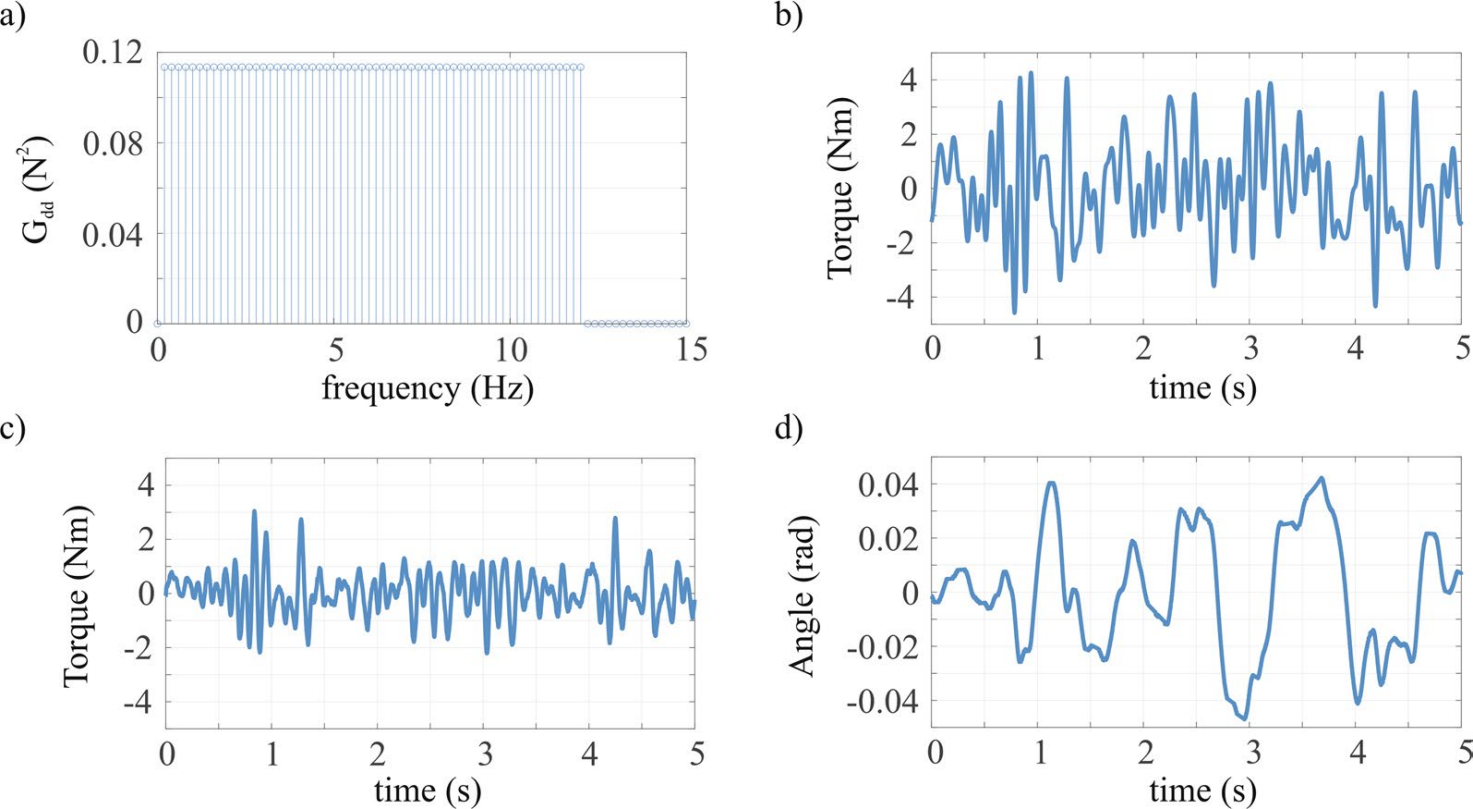
The torque perturbation signal in **a** frequency—and **b** time-domain was composed of frequencies between 0.2–12 Hz. **c** Typical elbow torque and **d** elbow angle throughout a single 5 s recording period

The torque perturbation signal was designed as a random-phase multisine torque signal (i.e., the sum of several sinusoids with random phase) to challenge the sensorimotor system with a frequency content relevant to study the dynamics of the elbow (natural frequency of the elbow is ~ 1–2 Hz). The period of the disturbance signal was set to 5 s to accommodate low frequencies but still enable sufficient periods in the given measurement time. All frequencies between 0.2 and 12 Hz were included in the perturbation signal (frequency resolution of 1/5 s = 0.2 Hz). The perturbation was scaled such to result in a root mean square elbow rotation during a trial of ~ 0.03 rad for each participant. Hence, each 55 s trial consisted of 11 consecutive periods of a perturbation period. The recorded elbow angle and torque were used to quantify elbow dynamics (Fig. 2).

To quantify protocol feasibility, for all participants, the total time required for the system identification protocol was noted and afterwards participants were asked to rate the pain of the protocol on a 0–10 scale (zero representing ‘no pain’). Moreover, forty participants (20 stroke patients and 20 healthy controls) were asked to rate the burden of the protocol on a 0–10 scale (zero representing ‘no burden’).

##### Re-Arm protocol

The results of the system identification protocol were compared with results obtained using the Re-Arm protocol presented previously [12, 20] to establish the construct validity of the system identification parameters. The Re-Arm protocol was designed to objectify manual performed clinical tests, thereby obtaining a comprehensive description of four characteristics that describe upper limb function: muscle weakness, abnormal synergy, spasticity, and viscoelasticity of the elbow. Three are used in this study:

1. To quantify abnormal synergy, participants were positioned to start at maximum elbow flexion and instructed to actively and slowly extend the elbow as far as possible. This was done once for five different levels of arm weight support (100%, 75%, 50%, 25%, and 0% support).
2. To quantify spasticity, participants were instructed to fully relax their arm and shoulder while the SEP passively moved their arm from maximum flexion to maximum extension at 100°/s. This was performed three times under full weight support of the arm and with a 5 s rest between repetitions.
3. To quantify viscoelasticity, participants were instructed to fully relax their arm and shoulder while the SEP passively moved their arm from maximum flexion to maximum extension and back at 6°/s. This was performed three times under full weight support of the arm and with a 5-s rest between repetitions.

### Data analysis

#### System identification analysis

Data from the system identification protocol was first analyzed using a non-parametric analysis to estimate elbow admittance (i.e., the resistance of the elbow to an external torque and thereby the inverse of impedance). The elbow admittance is represented by a frequency response function calculated by dividing the cross-spectral densities between the elbow angle ($\theta$) and elbow torque ($T$) with the perturbation torque ($d$):

$${H}_{T\theta }\left(f\right)= \frac{{S}_{d\theta }\left(f\right)}{{S}_{dT}\left(f\right)}$$

Cross-spectral densities ${S}_{d\theta }\left(f\right)$ and ${S}_{dT}\left(f\right)$ were estimated using Welch’s method [25] with a rectangular window corresponding to the length of the period in the perturbation signal (5 s). Before calculating the elbow admittance, the cross-spectral densities were smoothed using 3-point frequency averaging to reduce the variance of the estimations [26]. In addition to the elbow admittance, the coherence ${\widehat{\gamma }}_{\theta }^{2}(f)$ for the elbow angle was estimated following estimation of the auto-spectral densities ${S}_{\theta \theta }\left(f\right)$ and ${S}_{dd}\left(f\right)$:

$${\widehat{\gamma }}_{\theta }^{2}\left(f\right)= \frac{{\left|{S}_{d\theta }\left(f\right)\right|}^{2}}{{S}_{dd}\left(f\right){\cdot S}_{\theta \theta }\left(f\right)}$$

The coherence indicates whether the relation between perturbation torque and elbow angle is linear and noise-free.

Physiological meaningful parameters were obtained by fitting a neuromuscular model. This model represents the mechanical elbow admittance and the contact dynamics, i.e., dynamics associated with the interface between the lower arm and robotic lever. Therefore, the neuromuscular model is described by:

$${H}_{elbow}(s)=\frac{1}{I{s}^{2}+bs+k}+\frac{1}{{b}_{c}s+{k}_{c}}$$

where $I$ is the lower arm inertia, $b$ the elbow muscle viscosity, $k$ the elbow stiffness, ${b}_{c}$ the contact viscosity and ${k}_{c}$ the contact stiffness. The best model fit to the data was sought by minimizing the following criterion function [27]:

$$L\left(p\right)= \sum \frac{{\widehat{\gamma }}_{\theta }^{2}(f)}{1+f}{\left|ln\left(\frac{{H}_{T\theta }\left(f\right)}{{H}_{elbow}(f)}\right) \right|}^{2}$$

where we only included frequencies that had power in the perturbation signal following 3-point frequency averaging. A least-squares criterion with a logarithmic difference was used because of the large range in magnitude of the frequency response function. Moreover, the least-squares criterion was weighted with the coherence to limit the effect of the less reliable frequencies and with ${(1+f)}^{-1}$ to prevent emphasis on higher frequencies [27, 28]. Hence, both the DNI and resist conditions were fitted simultaneously with the same inertia. In addition, the contact dynamics were considered independent of the arm weight support level but different between the DNI and resist task conditions. For the DNI task, contact viscosity was taken as ${b}_{c}=2 Ns/m$ while contact stiffness was ${k}_{c}=340 N/m$, while for the resist task a contact viscosity of ${b}_{c}=4 Ns/m$ and contact stiffness of ${k}_{c}=500 N/m$ were used. These values provided an appropriate fit of the contact dynamics for all participants. In total, 15 parameters were estimated. Table 1 summarizes all model parameters to be estimated in this study.

**Table 1:** System identification parameters to be estimated

Parameter	Unit	Parameter	Unit
*Inertia*			
$I$	Nm·s^2^/rad		
*Viscosity*		*Elasticity*	
${b}_{100,DNI}$	Nm·s/rad	${k}_{100,DNI}$	Nm/rad
${b}_{75,DNI}$	Nm·s/rad	${k}_{75,DNI}$	Nm/rad
${b}_{50,DNI}$	Nm·s/rad	${k}_{50,DNI}$	Nm/rad
${b}_{25,DNI}$	Nm·s/rad	${k}_{25,DNI}$	Nm/rad
${b}_{0,DNI}$	Nm·s/rad	${k}_{0,DNI}$	Nm/rad
${b}_{100,res}$	Nm·s/rad	${k}_{100,res}$	Nm/rad
${b}_{0,res}$	Nm·s/rad	${k}_{0,res}$	Nm/rad

The subscripts indicate the percentage of arm weight that is supported (e.g. 100% is full arm weight support) and the task condition (DNI: ‘do not intervene’; res: resist)

Following fitting the neuromuscular model and extracting the system identification parameter values, two additional parameters, ${b}_{slope}$ and ${k}_{slope},$ were determined by performing a first-order polynomial least squares fit to the estimated elbow viscosity and stiffness across all weight support levels during the DNI task.

The goodness of the fit of the neuromuscular model, and thus quality of parametric estimates, was expressed using the variance-accounted-for (VAF) between the estimated and measured elbow angle. The estimated angle was calculated by multiplying the estimated mechanical admittance model with the perturbation torque in frequency domain, and subsequently taking the inverse Fourier transform. Any individual conditions were excluded from further analysis when the VAF < 0%, indicating a poor model estimate. When a participant had two or more missing DNI levels or a VAFs < 0%, the participant was excluded from further analysis.

#### Re-Arm protocol analysis

Data and results from the Re-Arm protocol were presented previously [20]. Here, we used the same data processing pipeline to extract synergy, spasticity, and viscoelasticity and thereby establish validity of the system identification technique:

1. To quantify abnormal synergy, the maximum elbow extension angle was determined for each level of weight support. The extension angle at 100% weight support was defined as zero. Subsequently, a linear regression line was estimated to relate weight support level to the maximum extension angle. The slope of the regression line was adopted as a measure of synergy, with 0 indicating an absence of synergy. A negative slope of the regression line would indicate the presence of synergy, since that would represent that the maximum elbow extension angle decreases with decreased arm weight support [29].
2. To quantify spasticity, the maximum torque during the three passive elbow extension movements was averaged.
3. To quantify viscoelasticity, the mean elbow torque at ten evenly-spaced positions during the ROM was extracted. The relationship between elbow angle and torque data was parameterized by fitting a regression line, of which the slope was taken as a measure of elbow viscoelasticity.

### Statistical analysis

The analysis was separated in four parts:

1. *Protocol feasibility and quality of the parametric estimates:* To establish feasibility of the system identification protocol and determine the quality of parametric estimates in patients we compared the total test time, perceived burden, pain score and quality of parametric estimates (using the VAF) between healthy controls and patients using an independent t-test or Mann–Whitney Test when data was non-normally distributed or had unequal variances across groups. Groups were considered statistically significantly different when p < 0.05.
2. *Test–retest reliability of the system identification parameters:* The test–retest reliability of the system identification parameters was determined by exploring Bland–Altman plots with the limits of agreement (LOA) (LOA = mean difference ± 1.96*standard deviation of the difference between the two measurements) and calculating intraclass correlation coefficients (ICCs). ICCs were calculated with two-way random effects, absolute agreement, single rater formula, ICC(2,1), where the ratio of the variance between participants to the variance between participants plus error variance was calculated. Values less than 0.4 indicated poor reliability, between 0.4 and 0.75, fair to good reliability, and higher than 0.75 are indicative of excellent reliability [30]. In addition to the ICC, the Smallest Detectable Change (SDC) was calculated as $SDC = 1.96 * SEM * \surd 2$ [31]. Different scores between two measurements larger than the SDC can be interpreted as true differences at an individual subject level.
3. *Healthy controls vs. patients:* We performed an exploratory analysis to test differences between groups in the mean and variance of the extracted system identification parameters. All 15 system identification parameters, and the two slope parameters, were treated as independent variables. Normality and equality of variances were tested using the Shapiro–Wilk test and Levene’s test for homogeneity of variances, respectively. Subsequently, a one-way ANOVA was performed to test for differences between groups. In case a parameter was found to be not normally distributed or presented unequal variance between groups, a Kruskal–Wallis test was performed. Any significant differences (p < 0.05) were further scrutinized using a (Dunn-)Bonferroni post hoc test.
4. *Construct validity of system identification parameters:* Construct validity was tested by comparing the parameters of the system identification protocol to the parameters extracted from the Re-Arm protocol. We used Pearson correlations to assess whether parameters from both protocols can be related. The following comparisons were made:
  *Synergy:* The system identification protocol does not involve any active movement, making a direct quantification of synergy by studying range of movement in different arm weight conditions as done routinely in clinical practice or during the Re-Arm protocol difficult. However, we assume that the abnormal synergy patterns causing impaired arm extension when reducing the arm’s weight support also results in a higher elbow viscosity and stiffness. This is based on observations that when reducing the arm’s weight support elbow extension is limited. The reduced reaching workspace has been suggested to be caused by an altered passive joint stiffness [32, 33]. Therefore, we correlated the change in elbow viscosity and stiffness across weight support levels ${({b}_{slope}, k}_{slope})$ with the slope of the regression line relating weight support level and maximum extension angle as obtained during the Re-Arm protocol.
  *Spasticity:* The system identification protocol does not administer fast passive elbow movements to quantify spasticity under full weight support of the arm. However, we assumed spasticity would become evident from the measures of elbow viscosity and stiffness when the elbow is fully relaxed and arm weight is fully supported during the DNI task which imposes small elbow movements at varying velocities. This assumption is based on the observation that spasticity is primarily reported to limit passive movement [34], while full arm weight support removes the potential confounding effect of synergy. Therefore, we correlated the elbow viscosity and elbow stiffness during the DNI task under full arm weight support ${({b}_{100,DNI}, k}_{100,DNI})$ with the Re-Arm parameter of spasticity (maximum torque during fast passive elbow extension).
  *Viscoelasticity:* The system identification protocol separates viscoelasticity, determined during slow passive elbow movement during full arm weight support, in elbow viscosity and stiffness. We assumed changes in viscoelasticity would be reflected in both elbow viscosity and elbow stiffness as determined during the DNI task under full arm weight support. Therefore, we correlated both the elbow viscosity and elbow stiffness during the DNI task under full weight support ${({b}_{100,DNI}, k}_{100,DNI})$ with the Re-Arm parameter of viscoelasticity (slope of the regression line relating elbow torque and angle).

The correlations coefficients were calculated using the *corrcoef* function in MATLAB2020a with the significance threshold set at $p<0.05.$

Statistical tests were conducted in SPSS version 25 for Windows (IBM, Armonk, NY, US) and values will be reported as mean (SD), unless stated otherwise.

### Sample size estimation

#### Sample size estimation for the test–retest reliability

Following Walter, Eliasziw [35], we tested whether the expected ICC (ρ1) was equal (null hypothesis) or higher (alternative hypothesis) than the acceptable ICC (ρ0). A ρ0 value of 0.60 was used, based on the literature, and a ρ1 of 0.85 [3, 36]. The number of observations was fixed at 2. Using a significance level (α) of 0.05 and a power (1–β) of 0.80, a sample size of 21 participants per group (patient and healthy control group) is required. To account for an expected dropout of 10%, the current study aimed at sample sizes of 24–30 participants who participated twice.

#### Sample size estimation for healthy controls vs. patients

We based our sample size calculation on the test of whether stiffness at a 100% weight support level was equal (null hypothesis) or different (alternative hypothesis) for the healthy controls and patients. Based on previous literature and preliminary findings we estimated the difference between the means to be $3\, \text{Nm/rad}$ with a standard deviation of $5\, \text{Nm/rad}$. Using a significance level (α) of 0.05 and a power (1–β) of 0.80, a sample size of 44 participants per group (patient and healthy control group) is required. To account for an expected dropout of 10%, the current study aimed at sample sizes of 45–50 participants.

#### Sample size estimation for the construct validity

We hypothesized that the system identification outcomes would correlate moderately (between 0.50 and 0.70) with their Re-Arm counterparts. Using a significance level (α) of 0.05 power (1–β) of 0.80 and an expected correlation coefficient of 0.50 a sample size of at least 29 participants per group (patient and healthy control group) is required. To account for an expected dropout of 10%, the current study aimed at sample sizes of at least 32 participants.

## Results

### Participants

In total, forty-five healthy controls, twenty-nine stroke patients, and twenty cerebral palsy patients participated. Twenty-five healthy controls, nine stroke patients, and all twenty cerebral palsy patients performed a second session of the measurement protocol to establish test–retest reliability. The characteristics of all three groups are summarized in Table 2.

**Table 2:** Characteristics of the groups of participants

	Healthy Controls (n = 45)	Patients (n = 49)	
		Stroke (n = 29)	CP (n = 20)
Gender (M/F)	15/30	23/6	9/11
Age (yrs)	51 (16)	64 (10)	40 (14)
Time post-stroke (months)	–	70 (35)	-
MACS level, median (range)	–	–	II (I-V)
GMFCS level, median (IQR)	–	–	II (I-V)
UE-FM scale, median (IQR) *	–	20 (12–24)	–
Partial TASC, median (IQR)	–	–	8 (5–8)
Modified Tardieu scale, quality of muscle reaction, median (range)	–	0 (0–5)	0 (0–5)

*n* number of subjects. *CP* Cerebral Palsy, *SD* standard deviation, *MACS* Manual Ability Classification System, *GMFCS* Gross Motor Function Classification System, *IQR* interquartile range, *Q* quality of muscle reaction (scale 0–5); MTS: Modified Tardieu Scale, *UE-FM* Upper Extremity—Fugl-Meyer assessment, *TASC* Test of Arm Selective Control; * Missing values for 20 subjects

### Protocol feasibility and quality of parametric estimates

The system identification protocol part of the experimental session took ~ 25 min (healthy controls: 25 (7.8) min; patients: 26 (7.5) min)—and was not different between the groups (U = 984.50, p = 0.37). The perceived burden of participation was scored similarly in 20 healthy controls 3.9 (2.5) and the 20 stroke patients 5.3 (2.3) (t(39) =  –1.86, p = 0.07). Pain for this protocol was also similar in the patients 1.5 (2.0) and healthy controls 0.7 (1.4) (U = 903.50, p = 0.09).

**Fig. 3:**
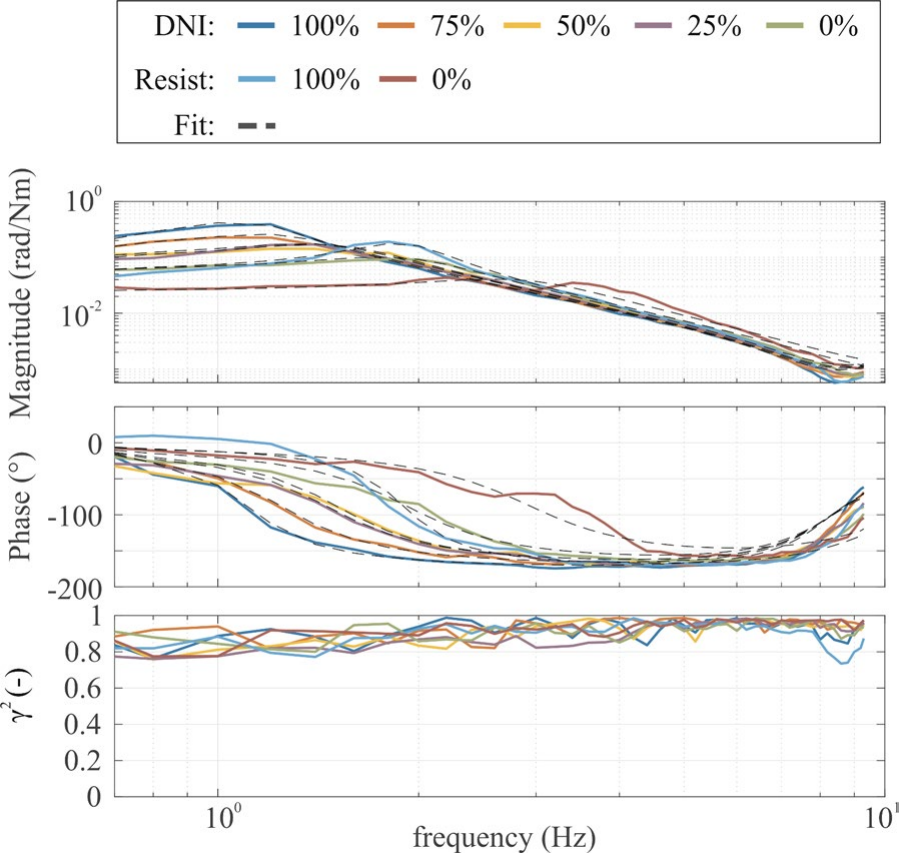
Frequency response functions magnitude, phase, and coherence for a typical stroke patient. Each colored line highlights a different level of arm weight support. The black dashed lines represent the best parametric fit for each condition. For this stroke patient, a lower elbow admittance magnitude (higher elbow stiffness) was found with reduced levels of arm weight support during the ‘do not intervene’ (DNI) task conditions, indicative of the presence of a shoulder-elbow synergy. Moreover, elbow admittance is lower during the active resist task than the DNI task. The high (> 0.8) coherence demonstrates a highly linear relationship between elbow torque and angle, and a low noise contribution

Figure 3 shows the magnitude and phase of the frequency response function and the coherence of a typical stroke patient for both the DNI and resist task and all levels of arm weight support used. In total, 9 of the 94 participants were excluded from further analysis (healthy controls: 6; patients: 3) due to poor (< 0%) VAFs and/or inability to successfully complete two or more levels. For the first measurement session, the fitted models (dashed lines) explained the underlying data well as highlighted by the VAF. Overall, the VAF was 79.3% (15.4%) for all conditions and participants, with no significant differences between healthy controls: 78.8% (16.8%) and patients: 79.7% (13.9%) (U = 45,854.0, p = 0.84).

### Test–retest reliability of the system identification parameters

**Fig. 4:**
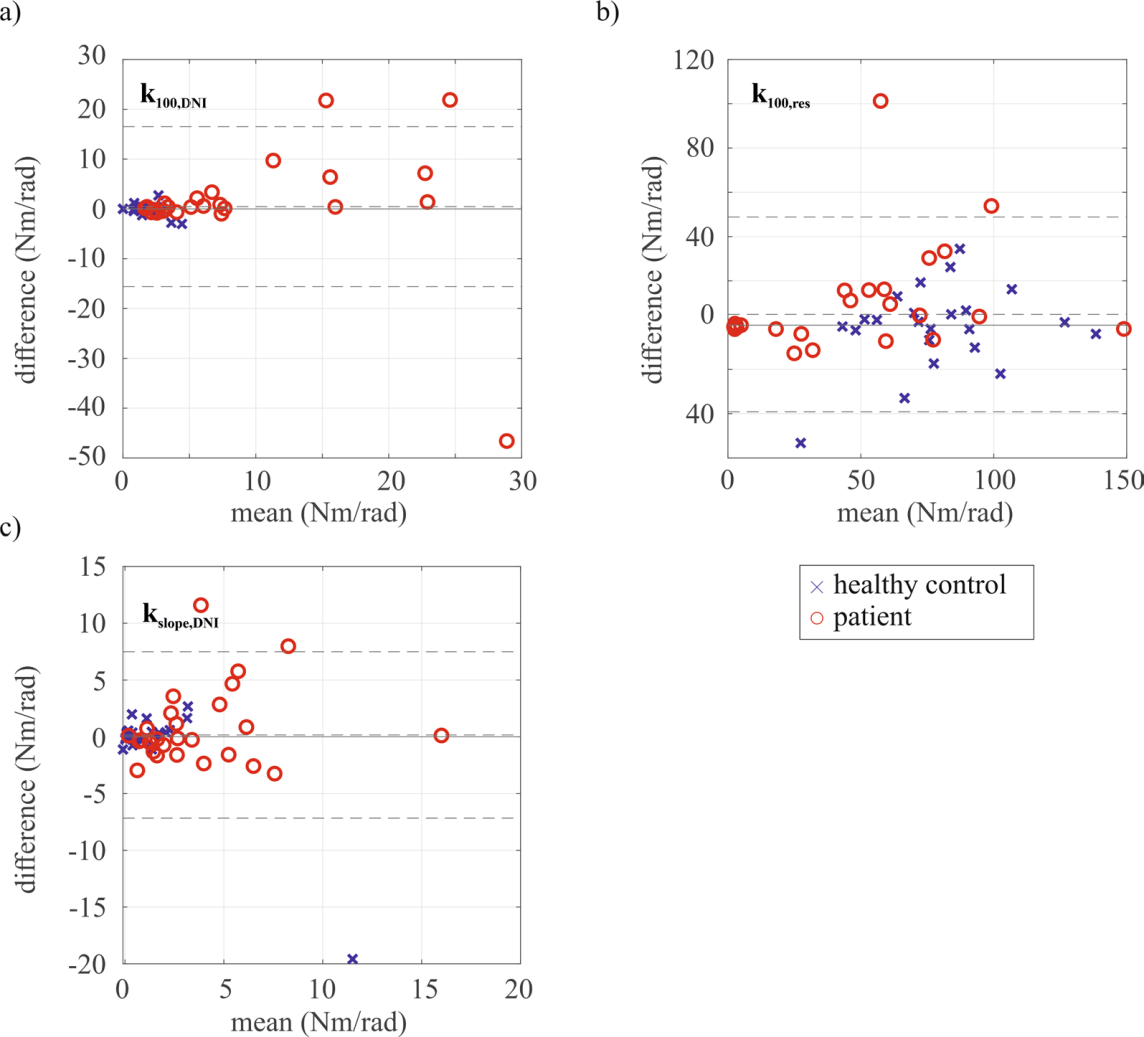
Bland–Altman plots for the parameters that are correlated with their corresponding Re-Arm parameters, including data from all participant groups. The mean and difference across a test and retest session for; **a** ${k}_{100,\mathrm{DNI}}$; **b** ${k}_{100,\mathrm{res}}$; and **c** ${k}_{\mathrm{slope}}$; are visualized. The mean difference and limits of agreement (mean_difference_ ± 1.96*SD_difference_) are indicated by the dashed horizontal lines

The Bland–Altman plots show the differences between test and retest for the system identification parameters that are correlated with their corresponding Re-Arm parameters: ${k}_{100,res}$, ${k}_{100,DNI}$ and ${k}_{slope}$ (Fig. 4). Overall, a fair to excellent test–retest reliability was found for the system identification parameters based on the ICC values (Table 3) for both healthy controls and patients. Poor reliability was found for elbow stiffness with full weight support in patients (${k}_{100,DNI}$), and the slope parameters together with elbow stiffness without weight support in healthy controls (${k}_{0,DNI}$, ${k}_{slope}$ and ${b}_{slope}$). The SDCs (Table 3) are higher for the patients group than the healthy controls.

**Table 3:** ICCs and SDCs for the system identification and slope parameters. ICC values less than 0.4 indicated poor reliability, between 0.4 and 0.75, fair to good reliability, and higher than 0.75 are indicative of excellent reliability

Parameter	ICC_healthy_	SDC_healthy_	ICC_patients_	SDC_patients_
$I$	0.98	0.008	0.94	0.014
${b}_{100,DNI}$	0.80	0.19	0.58	0.44
${b}_{75,DNI}$	0.73	0.51	0.69	0.69
${b}_{50,DNI}$	0.82	0.41	0.71	0.61
${b}_{25,DNI}$	0.91	0.27	0.63	0.70
${b}_{0,DNI}$	0.69	0.54	0.72	0.59
${b}_{100,res}$	0.93	0.52	0.59	1.88
${b}_{0,res}$	0.92	0.48	0.89	0.72
${b}_{slope}$	0.29	0.17	0.46	0.16
${k}_{100,DNI}$	0.55	2.24	0.35	22.10
${k}_{75,DNI}$	0.57	7.98	0.77	22.60
${k}_{50,DNI}$	0.80	8.91	0.82	24.78
${k}_{25,DNI}$	0.73	8.65	0.63	34.35
${k}_{0,DNI}$	0.22	28.26	0.83	22.27
${k}_{100,res}$	0.77	36.56	0.79	48.46
${k}_{0,res}$	0.86	26.75	0.80	37.14
${k}_{slope}$	0.13	7.99	0.58	6.61

### Differences between healthy controls and patients with upper limb impairments

**Fig. 5:**
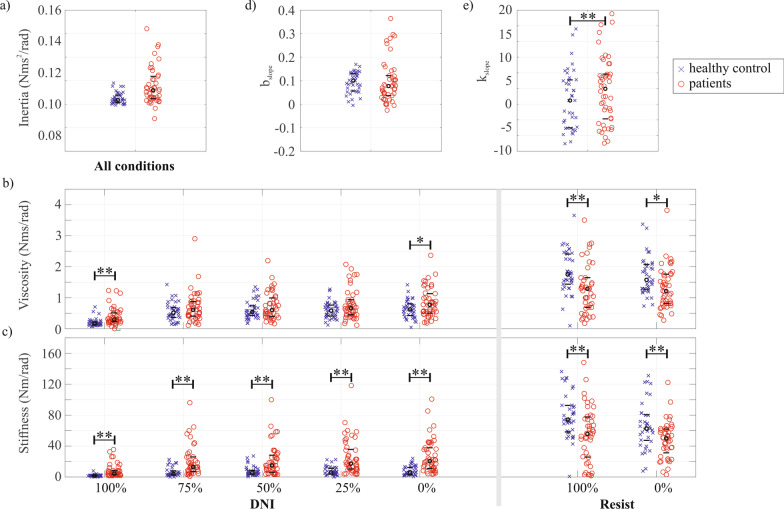
**a** Estimated elbow inertia; **b** estimated viscosity; and **c** estimated stiffness, for both tasks and all conditions, separated for healthy controls and patients. Joint inertia was kept constant across the tasks. **d** Change in viscosity (${b}_{slope}$); and **e** change in stiffness (${k}_{slope}$) across the ‘do not intervene’ conditions, separated by participant group. Each red or blue colored symbol marks one participant, whereas the black circle indicates the median and black dashes the 25th and 75th percentile.

Figure 5 shows an overview of the identified elbow inertia, elbow viscosity, elbow stiffness, and the stiffness and viscosity slope for all participants. The complete statistical results of the Levene’s and Mann–Whitney tests are presented in Additional file 1. Shapiro–Wilk test indicated that all parameters (all $$p < 0.05$$) presented a non-normal distribution, apart from $${k}_{0,res}$$ and $${k}_{100,res}$$. In addition, Levene’s test for homogeneity of variances indicated inequality of variance across groups for $${{b}_{100,DNI},{{b}_{50,DNI}, {b}_{25,DNI}, b}_{0,DNI}, {k}_{100,DNI}, {k}_{75,DNI}, {k}_{50,DNI}, {k}_{25,DNI}, {k}_{0,DNI}, k}_{slope}$$ and $${b}_{slope}$$ with all $$p < 0.05$$ (Additional file 1: Table S1). A significantly higher variance was found in the patients compared to the healthy controls for all but one ($${b}_{slope})$$ parameter (Additional file 1: Table S2). As a result, any differences between groups for each parameter were explored using a nonparametric Mann–Whitney test.

For the DNI task, elbow viscosity and stiffness where higher in the patients than the healthy controls in ${b}_{100,DNI},{ b}_{0,DNI}, {k}_{100,DNI}, {k}_{75,DNI}, {k}_{50,DNI}, {k}_{25,DNI}$ and ${k}_{0,DNI}$ with all $p<0.05$ (Additional file 1: Table S3). Moreover, for all parameters during the resist task conditions except ${b}_{0,res}$ we found a lower elbow viscosity and stiffness for the patients compared to the healthy controls (all $p<0.01$). Finally, ${k}_{slope}$ was significantly higher in the patients than the healthy controls ($p<0.01$) whereas elbow inertia $(I)$ and change in elbow viscosity across arm weight support levels $({k}_{slope})$ did not significantly differ between participant groups ($I$: $p=0.06$; ${k}_{slope}$: $p =0.40$).

### Construct validity of system identification parameters

**Fig. 6:**
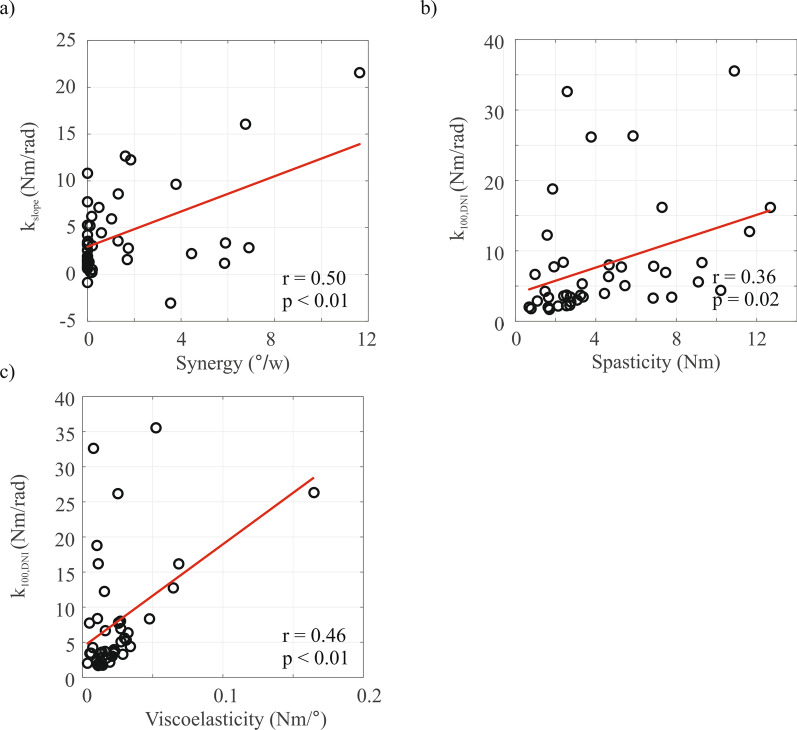
Correlation between parameters extracted using the Re-Arm and system identification protocol, only data from the include patients is used: **a** synergy vs. ${k}_{slope}$; **b** spasticity vs. ${k}_{100,\mathrm{DNI}}$ and **c** viscoelasticity vs. ${k}_{100,\mathrm{DNI }}.$ Significant, weak to moderate, correlations were found between the Re-Arm parameters and corresponding stiffness parameters extracted using system identification

The Re-Arm parameters are visualized against the system identification and slope parameters of the same participants in Fig. 6. Firstly, no significant correlation was found between synergy and viscosity slope ${b}_{slope}$ $(r=0.06, n=46, p=0.69)$, but a moderate positive correlation was found with stiffness slope ${k}_{slope}$ $(r=0.50, n=46, p< 0.01)$. Secondly, statistically significant, positive but moderate correlations were found between the spasticity parameter and elbow viscosity and stiffness $({b}_{100,DNI}: r=0.39, n=44, p=0.01; {k}_{100,DNI}: r=0.36, n=44, p=0.02)$. Finally, a moderate and significant correlation was found between viscoelasticity and the elbow viscosity and elbow stiffness during the DNI task under full arm weight support $({b}_{100,DNI}: r=0.35, n=44, p=0.02; {k}_{100,DNI}: r=0.46, n=44, p<0.01)$.

## Discussion

In this study, we aimed to establish the merits of quantifying abnormal synergy, spasticity and changes in joint viscoelasticity using system identification. Feasibility was confirmed by demonstrating that the experimental protocol could be completed by all participants in ~ 25 min without pain or burden. Moreover, the quality of the parametric estimates was good, with VAFs of ~ 80%. Test–retest reliability for most system identification parameters was fair to excellent ($ICC = 0.46-0.98$), for both healthy controls and patients, except for elbow stiffness with no weight support for the controls $(ICC = 0.22)$ and full weight support for the patients ($ICC = 0.35$). Moreover, the slope parameters describing the change in elbow stiffness or viscosity with reduced arm weight support showed a poor reliability for healthy controls $(ICC- {b}_{slope}=0.29, {k}_{slope}=0.13)$ and fair reliability for patients $\left(ICC - {b}_{slope}=0.46, {k}_{slope}=0.58\right).$ Overall, the system identification results demonstrate higher elbow viscosity and stiffness within patients than the healthy controls during the DNI task conditions, and lower elbow viscosity and stiffness during the resist task conditions. The system identification and slope parameters presented a weak to moderate, but significant, correlation with their matching parameters as determined using the Re-Arm protocol ($r = 0.36-0.50$ with $p < 0.03$). Taken together, this work demonstrates that system identification is a feasible and reliable way to quantify upper limb motor impairments. Validity was confirmed by differences between patients and controls and correlations with other measurements, but further work is required to optimize the experimental protocol and establish clinical value.

### Feasibility of the system identification protocol

This study demonstrated the feasibility of obtaining data that enables quantifying upper limb impairments using system identification with a limited time interval of ~ 25 min and without significant pain or burden. Moreover, both for the healthy control and patient group, the parametric fits of the frequency response functions used to extract parameters describing the elbow joint dynamic proved to be of good quality with a VAF of ~ 80%. The time required to perform the system identification protocol is comparable to the time used to administer the Re-Arm protocol (~ 30 min), but longer than conventional clinimetry such as a combination of the upper extremity part of the FMA and the MAS (in total ~ 10 min). Whereas in the current study, only stroke and CP patients were included, the measurement setup does enable assessment of other patient groups, e.g., multiple sclerosis, and spinal cord injury, within the provided inclusion and exclusion criteria. Future work will need to establish whether it is possible to reduce the time required to obtain all data while retaining, and improving, the reliability and validity of the extracted clinical information.

### Test–retest reliability of the system identification parameters

The major criticism towards clinical scales such as the FMA, MAS and Tardieu scale is their poor validity, reliability, sensitivity, and inability to isolate concomitant phenomena like spasticity and change in viscoelastic properties. Yet, for well-trained assessors, the inter- and intra-rater reliability is fair to excellent with ICCs ranging from 0.7–0.8 [3, 5]. Generally, fair to excellent intrasubject reliability can also be achieved for system identification parameters [16, 37, 38], although this was mostly explored in small populations of healthy participants. The order of magnitude of these ICCs is like those obtained with comparable measurements in joints other than the elbow ($ICC = 0.64-0.91$)[15, 39]. In this study, the test–retest reliability of the viscosity and stiffness, the primary parameters we use to quantify the motor impairments, ranges from poor to excellent $ICC=0.22-0.93$ with poor reliability only found for the slope parameters and ${k}_{0,DNI}$ in healthy controls and ${k}_{100,DNI}$ in patients.

Specifically for the measures used to evaluate synergy $${(k}_{slope})$$ and spasticity ($${k}_{100,DNI})$$, the reliability was poor to fair. Evaluation of the presence of synergy is performed by a linear fit of elbow stiffness across all weight support levels of the arm. This ‘slope’ parameter presented a fair reliability (ICC = 0.58), which is lower than the reliability found for the synergy score obtained using the Re-Arm protocol (ICC = 0.78). The difference may be caused by inappropriate task performance or fatigue which is more likely during the 55 s trials performed in this study. No other studies have reported test–retest reliability using the ICC of a measure for upper limb muscle synergies. Spasticity was quantified using the elbow stiffness when the elbow is fully relaxed and arm weight is fully supported ($${k}_{100,DNI})$$. This measure had a poor reliability ($$ICC=0.35$$) for patients, in contrast to the excellent reliability $$\left(ICC = 0.95\right)$$ found for the spasticity measure using the Re-Arm protocol. For spasticity, other robotic measurement instruments also reported higher ICCs between 0.66–0.95 in stroke patients [39–41].

Hence, the lowest reliability in patients was found for the tasks where the full weight of the arm was supported. This suggests the patients have greater difficulty consistently controlling the elbow joint without having to worry about supporting their weight by actively lifting the arm. Although this was not directly evident when the experiments were performed, this should be considered in future studies. The reliability of the system identification parameters may improve by collecting additional data within a participant, now limited to 55 s of data per arm weight support level, but this comes at the cost of patient comfort and protocol duration.

### Differences in elbow dynamics between healthy controls and patients with an upper limb impairment

Upper limb impairments are often associated with significantly higher joint resistance to movement, which may be caused by changes in both active and passive muscle properties [39, 42]. The resistance can be quantified by measuring the resistive torque during passive movement and can be separated into contributions from changes in viscosity and stiffness. In this study, we further support previous findings of changes in joint resistance, using system identification to reveal an increased viscosity and stiffness during the passive DNI task conditions and decreased viscosity and stiffness during the active resist task conditions for a subset of the stroke and CP patients.

Altered elbow dynamics may also be caused by muscle synergies and spasticity. Upper limb synergy is a commonly reported upper limb impairment and is characterized by an involuntary coupling of shoulder abduction and elbow flexion, resulting in reduced reaching capacity [13, 33, 43, 44]. We revealed an increase in elbow stiffness with reduced arm weight support in the patient group, not present in the healthy controls. This finding explains the reduced reaching capacity under the same task when reducing arm weight support as reported by others [13, 32]. Spasticity, in our data may explain the enhanced elbow stiffness when the elbow is fully relaxed and arm weight is fully supported. An increased reflexive activity in upper limb impairments due to e.g. stroke and in CP [14, 45, 46], may result in an enhanced reflexive stiffness and thereby contribute to the an increased overall stiffness. Together, the broad number of differences across different system identification parameters show the heterogeneity of the patient population, highlighting the importance of adequate quantification of the present impairments to successfully direct treatment.

### Construct validity of the system identification parameters to quantify upper limb motor impairments

The adopted measures to quantify synergy, spasticity, and viscoelasticity of the elbow joint correlated moderately ($$r = 0.35-0.50)$$ but significantly with their Re-Arm counterparts. Clinically, and in the Re-Arm protocol, synergy, spasticity, and viscoelasticity are determined while the participant’s elbow is passively or actively moving across its full ROM. In this study, the measures used to quantify synergy and spasticity are obtained during a passive postural task, and as a result, the moderate correlations may suggest that an active voluntary movement may not be necessary to quantify synergy or spasticity when the participant is unable to perform these due to, e.g., pain. However, motor impairments such as synergies and spasticity may not be adequately revealed during a postural task at a single joint such as performed in the current study, and therefore correlations should be interpreted with care, especially since they are moderate and use a relatively small sample sizes.

During the system identification protocol, the joint dynamics are characterized while participants keep a fixed posture, and a torque perturbation results in small elbow rotations. Keeping a fixed posture for system identification is required to enable the application of time-invariant system identification, as during movement, there is a profound modulation of intrinsic and reflexive properties [47–50]. A more complete image of how joint dynamics is modulated over the ROM can be obtained by performing the same postural task at multiple joint angles [51–54], but this does not reveal any impairments only expressed during active movement. Quantifying motor impairments during movement using system identification requires the application of time-varying system techniques [55]. Unfortunately, these techniques have yet to be applied in the study of motor impairments due to their complex data requirements and lack of clinical scope of their outcome parameters.

Specifically for spasticity, the use of a more extensive parametric model that also quantifies reflexive stiffness, rather than a compound measure of elbow stiffness, may be expected to have a stronger correlation with spasticity than the moderate correlation found. System identification enables separating joint stiffness in their intrinsic and reflexive contributions with or without recording electromyography from the muscles involved [18, 19]. Alibiglou, Rymer [51] did this but found no significant correlations between either intrinsic or reflexive stiffness and the MAS for stroke patients. In this study, reflexive stiffness was not quantified as the 55 s of data collected is insufficient to obtain a reliable estimate for the reflexive stiffness. A different perturbation signal, such as a pseudorandom binary sequence perturbation may be better suited to assess reflexive joint properties.

## Conclusions

This work demonstrates that system identification is a feasible way to quantify upper limb motor impairments. By acquiring data during only two distinct tasks, one active and one passive task, system identification provides a complete description of the joint dynamics within half an hour. Pronounced differences between healthy controls and patients can be identified while the reliability and validity of the extracted parameters is moderate. Changes in joint dynamics can be related to traditional motor impairments like synergy, spasticity, and viscoelasticity. Even though robotics has enabled objective quantification of upper limb impairments, the use of robotics for diagnosis has yet to make its way into routine clinical practice. An important reason for the lack of clinical use is that very few studies introducing new diagnostic measures obtained using robotics make their way past providing a correlation with current routine clinical scores or comparing patients and healthy controls [56]. The latter comparison may not be appropriate given motor impairments are not always clearly defined and clinical scores may be unreliable and insensitive. More work is needed to provide evidence that measures such as extracted using system identification, provide clinically meaningful outcome measures that are more reliable and less time-consuming than current clinical scales.

## Supplementary Information

**Additional file 1. Tables S1:** Results of the Levene’s tests of homogeneity in variance. **Table S2:** Results of the Mann-Whitney tests on the group variance.** Table S3:** Results of the Mann-Whitney tests.

## Data Availability

The dataset supporting the conclusions of this article is available from the corresponding author upon reasonable request.

## Abbreviations

CP: Cerebral Palsy
DNI: Do Not Intervene
GMFCS: Gross Motor Function Classification System
ICC: Intraclass Correlation Coefficient
IQR: Interquartile Range
MACS: Manual Ability Classification System
MAS: Modified Ashworth Scale
MTS: Modified Tardieu Scale
MVT: Maximum Voluntary Torque
SD: Standard Deviation
SEP: Shoulder Elbow Perturbator
TASC: Test of Arm Selective Control
UE-FM: Upper Extremity-Fugl-Meyer
VAF: Variance Accounted For
