# Supplementary material for: System identification: a feasible, reliable and valid way to quantify upper limb motor impairments
Source: J Neuroeng Rehabil. 2023 May 25;20:67. doi: 10.1186/s12984-023-01192-x (PMC10210505; doi:10.1186/s12984-023-01192-x)
Supplement: Supplementary file 1 — Additional file 1. Tables S1: Results of the Levene’s tests of homogeneity in variance. Table S2: Results of the Mann-Whitney tests on the group variance. Table S3: Results of the Mann-Whitney tests. [file 12984_2023_1192_MOESM1_ESM.docx]

**Additional file 1**

**Tables S1:** Results of the Levene’s tests of homogeneity in variance.

| **Parameter** | **F** | **df1** | **df2** | $\boldsymbol{p}$**^*^** |
| --- | --- | --- | --- | --- |
| $I$ | 1.04 | 1 | 91 | 0.31 |
| $b_{100,DNI}$ | 16.96 | 1 | 89 | **0.00** |
| $b_{75,DNI}$ | 2.40 | 1 | 84 | 0.13 |
| $b_{50,DNI}$ | 4.26 | 1 | 88 | **0.04** |
| $b_{25,DNI}$ | 8.39 | 1 | 88 | **0.01** |
| $b_{0,DNI}$ | 6.33 | 1 | 89 | **0.01** |
| $b_{100,res}$ | 2.75 | 1 | 86 | 0.10 |
| $b_{0,res}$ | 0.65 | 1 | 86 | 0.42 |
| $k_{100,DNI}$ | 34.97 | 1 | 89 | **0.00** |
| $k_{75,DNI}$ | 19.29 | 1 | 83 | **0.00** |
| $k_{50,DNI}$ | 24.65 | 1 | 88 | **0.00** |
| $k_{25,DNI}$ | 28.72 | 1 | 88 | **0.00** |
| $k_{0,DNI}$ | 30.06 | 1 | 89 | **0.00** |
| $k_{100,res}$ | 2.41 | 1 | 86 | 0.12 |
| $k_{0,res}$ | 0.45 | 1 | 86 | 0.50 |
| $b_{slope}$ | 8.82 | 1 | 79 | **0.00** |
| $k_{slope}$ | 18.75 | 1 | 79 | **0.00** |

^*^ Bold $p$-values are significant at a $p<0.05$ level.

**Table S2:** Results of the Mann-Whitney tests on the group variance.

| **Parameter^§^** | $\boldsymbol{p}$**^*^** |  | **Parameter** | $\boldsymbol{p}$**^*^** |
| --- | --- | --- | --- | --- |
| $\boldsymbol{b}_{\mathbf{100,}\boldsymbol{DNI}}$ | **0.00** |  | $\boldsymbol{k}_{\mathbf{25,}\boldsymbol{DNI}}$ | **0.00** |
| $\boldsymbol{b}_{\mathbf{50,DNI}}$ | **0.04** |  | $\boldsymbol{k}_{\mathbf{0,}\boldsymbol{DNI}}$ | **0.00** |
| $\boldsymbol{b}_{\mathbf{25,DNI}}$ | **0.03** |  | $\boldsymbol{b}_{\boldsymbol{slope}}$ | 0.07 |
| $\boldsymbol{b}_{\boldsymbol{0}\mathbf{,DNI}}$ | **0.02** |  | $\boldsymbol{k}_{\boldsymbol{slope}}$ | **0.00** |
| $\boldsymbol{k}_{\mathbf{100,}\boldsymbol{DNI}}$ | **0.00** |  |  |  |
| $\boldsymbol{k}_{\mathbf{75,}\boldsymbol{DNI}}$ | **0.00** |  |  |  |
| $\boldsymbol{k}_{\mathbf{50,}\boldsymbol{DNI}}$ | **0.00** |  |  |  |

**^§^** for significant Levene’s tests of homogeneity in variance results only.

^*^ Bold $p$-values are significant at a $p<0.05$ level.

**Table S3:** Results of the Mann-Whitney tests.

| **Parameter** | **U** | $\boldsymbol{p}$**^*^** |
| --- | --- | --- |
| $I$ | 836.00 | 0.06 |
| $b_{100,DNI}$ | 372.00 | **0.00** |
| $b_{75,DNI}$ | 742.00 | 0.12 |
| $b_{50,DNI}$ | 855.00 | 0.21 |
| $b_{25,DNI}$ | 809.00 | 0.10 |
| $b_{0,DNI}$ | 773.00 | **0.04** |
| $b_{100,res}$ | 577.00 | **0.00** |
| $b_{0,res}$ | 628.00 | **0.01** |
| $k_{100,DNI}$ | 171.00 | **0.00** |
| $k_{75,DNI}$ | 370.00 | **0.00** |
| $k_{50,DNI}$ | 477.00 | **0.00** |
| $k_{25,DNI}$ | 347.00 | **0.00** |
| $k_{0,DNI}$ | 332.00 | **0.00** |
| $k_{100,res}$ | 610.00 | **0.00** |
| $k_{0,res}$ | 589.00 | **0.00** |
| $b_{slope}$ | 716.00 | 0.40 |
| $k_{slope}$ | 403.00 | **0.00** |

^*^ Bold $p$-values are significant at a $p<0.05$ level.
